# An UPLC-MS/MS Assay to Measure Glutathione as Marker for Oxidative Stress in Cultured Cells

**DOI:** 10.3390/metabo9030045

**Published:** 2019-03-05

**Authors:** Katharina Herzog, Lodewijk IJlst, Arno G. van Cruchten, Carlo W.T. van Roermund, Wim Kulik, Ronald J. A. Wanders, Hans R. Waterham

**Affiliations:** 1 Centre for Analysis and Synthesis, Department of Chemistry, Lund University, 223 62 Lund, Sweden; katharina.herzog@chem.lu.se; 2 Laboratory Genetic Metabolic Diseases, Department of Clinical Chemistry, Amsterdam UMC, Location AMC, University of Amsterdam, 1105 AZ Amsterdam, The Netherlands; l.ijlst@amc.uva.nl (L.I.); a.vancruchten@amc.uva.nl (A.G.v.C.); c.vanroermund@amc.uva.nl (C.W.T.v.R.); w.kulik@amc.uva.nl (W.K.); r.j.wanders@amc.uva.nl (R.J.A.W.)

**Keywords:** oxidative stress, glutathione, cultured cells, yeast, mass spectrometry

## Abstract

Oxidative stress plays a role in the onset and progression of a number of diseases, such as Alzheimer’s disease, diabetes and cancer, as well as ageing. Oxidative stress is caused by an increased production of reactive oxygen species and reduced antioxidant activity, resulting in the oxidation of glutathione. The ratio of reduced to oxidised glutathione is often used as a marker of the redox state in the cell. Whereas a variety of methods have been developed to measure glutathione in blood samples, methods to measure glutathione in cultured cells are scarce. Here we present a protocol to measure glutathione levels in cultured human and yeast cells using ultra-performance liquid chromatography-tandem mass spectrometry (UPLC–MS/MS).

## 1. Introduction

The interest in studying oxidative stress mechanisms at the cellular level is increasing, primarily because of the current understanding that oxidative stress plays a role in the onset and progression of ageing and a variety of human diseases, including Alzheimer’s disease, diabetes and cancer [1,2,3]. Besides mouse models, in vitro approaches using cultured cells and model organisms are widely used to manipulate and study the cellular response to oxidative stress triggers. Among the different indicators of oxidative stress, glutathione (L-γ-glutamyl-cysteinyl-glycine) is often studied because of its presence in high concentrations in nearly all cell types. Glutathione exists in a reduced form (GSH) and, as disulphide, in an oxidised form (GSSG) [2,3], and the GSH/GSSG ratio is often used as a marker of the redox state in the cell [1]. Like other thiols, however, glutathione is highly reactive towards radicals, and auto-oxidation of GSH easily occurs during sample preparation [4]. It has been reported that up to 3% of GSH is oxidised in cultured cells and physiological fluids such as blood during sample preparation, resulting in a dramatic increase of GSSG [4,5]. Of note, all reported methods greatly vary in sample preparation, especially in the derivatisation and deproteinisation of samples [4,5,6]. Several methods have been developed to circumvent auto-oxidation, providing remarkable differences in reported concentrations of GSH and GSSG. Acids especially, which are commonly used for deproteinisation, are known to induce oxidation of GSH by forming hydrogen peroxide [4,5]. Another factor is the reduction of GSSG to GSH by glutathione reductase during sample preparation, which may occur when the enzymatic activity is not blocked immediately. Furthermore, whereas several methods determine the total glutathione content by reducing GSH to GSSG using an artificial oxidant, other methods measure GSH and GSSG directly [1]. Consequently, a standardised protocol is needed for sample preparation, with the overall goal to make glutathione concentrations comparable [3]. Here we present a protocol using ultra-performance liquid chromatography-tandem mass spectrometry (UPLC–MS/MS) and the combination of N-ethylmaleimide (NEM) and acetonitrile for rapid derivatisation and deproteinisation of samples as a suitable method to study glutathione in cultured cells. The use of NEM, an alkylating agent for thiols, is ideal for preventing auto-oxidation, because of its fast action mechanism on thiols. In addition, NEM inhibits the activity of glutathione reductase [2,4], which contributes to the accurate measurement of the GSH/GSSG ratio. By measuring GSH and GSSG concentrations in cultured cells, including human and yeast cells, this protocol allows the study of the redox state in cells and their susceptibility to oxidative stress.

## 2. Experimental Design

**Figure 1:**
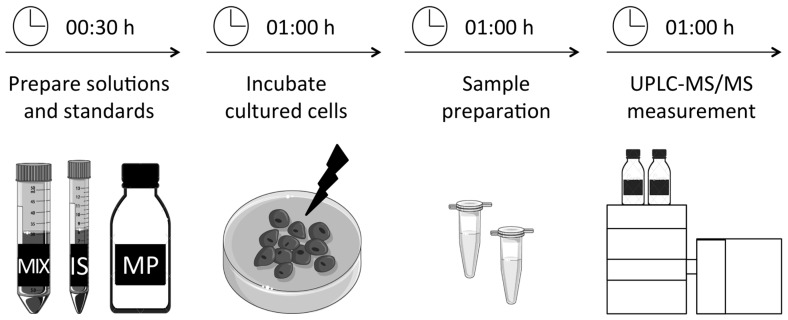
Graphical overview summarising the experimental setup and time schedule.

The cells were incubated for the indicated times (Figure 1; see the Procedure section for details of experiments). To harvest the cells, we used a precipitation mix that contained ice-cold acetonitrile and N-ethylmaleimide (NEM), which was added to the cells immediately after removal of the culture medium, together with the labelled internal standard. We collected the cells by scraping and centrifuged the mixture. The supernatant was evaporated, and we dissolved the residue in the UPLC mobile phase. For instant measurement of the redox state in the cell, we used NEM in combination with acetonitrile for immediate deproteinisation to block all enzymatic activities and to mask the thiol groups.

For each analysis, a calibration curve using serial diluted amounts of unlabelled analyte and a constant amount of a corresponding isotopically labelled internal standard (see Section 3.1 for details) was prepared to allow for the calculation of the concentration of GSH and GSSG in the sample. In particular, serial diluted amounts of unlabelled analyte for GSSG ranging from 0–3.25 µM, and GSH-NEM ranging from 0–32.41 µM were used for the generation of calibration curves. Depending on the application, the user may consider adapting the points in the calibration curve based on the range of expected values for GS-NEM and GSSG.

To minimise auto-oxidation during sample preparation, it is important to add both NEM and acetonitrile to the cells immediately after removal of the culture medium, without prior washing steps. We highly recommend the use of appropriate positive controls, i.e., cells that underwent an oxidative challenge by incubation with chemicals such as menadione and buthionine sulfoximine (BSO) (see the Procedure section for details). Similarly, we recommend the use of appropriate negative controls, i.e., cells with vehicle treatment using equal amounts of solvents used for incubation with chemicals. In addition, we recommend conducting every experiment at least in triplicate.

See Appendix B for a comparison of results with those obtained by other LC-MS-based protocols.

### 2.1. Materials

L-Glutathione reduced (C_10_H_17_N_2_NO_6_S), ≥98.0% peptide purity (Sigma-Aldrich, St. Louis, MO, USA; Cat.no.: G4251)

L-Glutathione oxidized (C_20_H_32_N_6_O_12_S), ≥98.0% peptide purity (Sigma-Aldrich, St. Louis, MO, USA; Cat.no.: G4376)

Labelled reduced glutathione-(glycine-^13^C_2_,^15^N) (C_8_^13^C_2_H_17_N_2_^15^NO_6_S), ≥98 atom % ^15^N, ≥99 atom % ^13^C, ≥95% (CP) (Sigma-Aldrich, St. Louis, MO, USA; Cat.no.: 683620)

N-ethylmaleimide (NEM), ≥98.0% peptide purity (Sigma-Aldrich, St. Louis, MO, USA; Cat.no.: E3876)

DL- Buthionine-(S,R)-sulfoximine (BSO) (Sigma-Aldrich, St. Louis, MO, USA; Cat.no.: B2640)

Menadione (Sigma-Aldrich, St. Louis, MO, USA; Cat.no.: M5625)

Dimethyl sulfoxide (DMSO) (Sigma-Aldrich, St. Louis, MO, USA; Cat.no.: 673439)

Acetonitrile (99.9%) (Biosolve BV, Valkenswaard, The Netherlands; Cat. no.: 0001203502BS)

Ethanol absolute (Dehydrated) AR (Biosolve BV, Valkenswaard, The Netherlands; Cat. no.: 052505)

Formic acid (98–100%) (Merck, Darmstadt, Germany; Cat. no.: 1.00264.1000

Hydrogen peroxide (30%) (Merck, Darmstadt, Germany; Cat. no.: 1.07209.0250

Potassium iodide (ISO grade) (Merck, Darmstadt, Germany; Cat. no.: 105043)

Natrium hydroxide (VWR Chemicals, Radnor, Pennsylvania, USA; Cat.no.:28244.295)

Ultrapure water (Milli-Q) (Merck, Darmstadt, Germany; Cat. no.: Milli-Q^®^ IQ 7000)

Corning Costar Flat Bottom Cell Culture Plates (6 well format) (Sigma-Aldrich, St. Louis, MO USA; Cat.no.: CLS3736)

Corning Cell scrapers (Sigma-Aldrich, St. Louis, MO, USA; Cat.no.: CLS3010)

Eppendorf Safe-Lock Tubes, 2.0 mL (Eppendorf, Hamburg, Germany; Cat. no.: 0030120094)

### 2.2. Equipment

Refrigerated microcentrifuge suitable for 2.0 mL tubes (Centrifuge 5425R) (Eppendorf, Germany; Cat. no.: 5404000014)

Dri-block (sample concentrator), Techne DB-3 (Staffordshire, UK)

Acquity UPLC HSS T3 1.8 µm column (2.1 × 100 mm) (Waters, Massachusetts, USA; Cat. no.: 186003539)

Acquity UPLC system consisting of an Acquity Solvent Manager with degasser and an Acquity Sample Manager with column oven (Waters, Milford, MA, USA)

Masslynx V4.1 software (Waters, Milford, MA, USA)

Quattro Premier XE triple quad (Waters, Milford, MA, USA), serial no.: VAB964

### 2.3. Cultured Primary Fibroblasts

We used anonymised primary skin fibroblast cell lines from healthy controls, Zellweger Spectrum Disorder patients with a severe phenotype (homozygous for the severe c.2097insT mutation in *PEX1*), and patients diagnosed with 5-oxoprolinuria due to 5-oxoprolinase deficiency and glutathione synthetase-deficient cell lines which were obtained from Coriell Cell Repositories (Camden, NJ, USA). Fibroblasts were cultured in 25-cm^2^ flasks in Ham’s F-10 Medium with L-glutamine, supplemented with 10% foetal calf serum (Invitrogen, Carlsbad, CA, USA), 25 mM Hepes, 100 U/mL penicillin and 100 µg/mL streptomycin and 250 µg/mL amphotericin in a humidified atmosphere of 5% CO_2_ at 37 °C.

### 2.4. Saccharomyces Cerevisiae

We used the strain BJ1991 (MATα, leu2, trp1, ura3-251, prb1-1122, pep4-3, gal2) as wild-type strain. The *glr1* deletion strain was generated via replacement of the entire *GLRI* open reading frame by the *kanMX* gene as described previously [6]. Wild-type and deletion strains were cultured in minimal medium containing 6.7 g/L yeast nitrogen base (YNB-WO) and supplemented with 5 g/L glucose or 20 g/L ethanol and amino acids (20 µg/mL) as needed.

## 3. Procedure

### 3.1. Preparation of Solutions and Standards. Time for Completion: 00:30 Hours

1. Prepare the NEM stock solution freshly on the day of experiment. Dissolve NEM in Milli-Q water to a final concentration of 20 mM.
  When using *S. cerevisiae*, prepare wash solutions containing NEM in Milli-Q water. Per sample, prepare 300 µL of wash solution containing 200 mM NEM, and 2 mL of wash solution containing 50 mM NEM.
2. Prepare the precipitation solution freshly on the day of the experiment. Per sample, the precipitation solution consists of 280 µL NEM and 700 µL acetonitrile. Cool on ice prior to use.
3. Prepare the internal standard mix. Per sample, the internal standard mix contains 10 µL labelled GSH (stock solution 650 µM, in Milli-Q) and 10 µL labelled GSSG (stock solution 3.25 µM, in Milli-Q) (see Section 5. Reagents Setup for details). Cool on ice prior to use.
4. Prepare the mobile phases for the UPLC system. Mobile phase A consists of 0.1% formic acid in water, mobile phase B consists of 0.1% formic acid in acetonitrile.

### 3.2. Incubation and Preparation of Primary Fibroblasts. Time for Completion: 02:00 Hours

**Figure 2:**
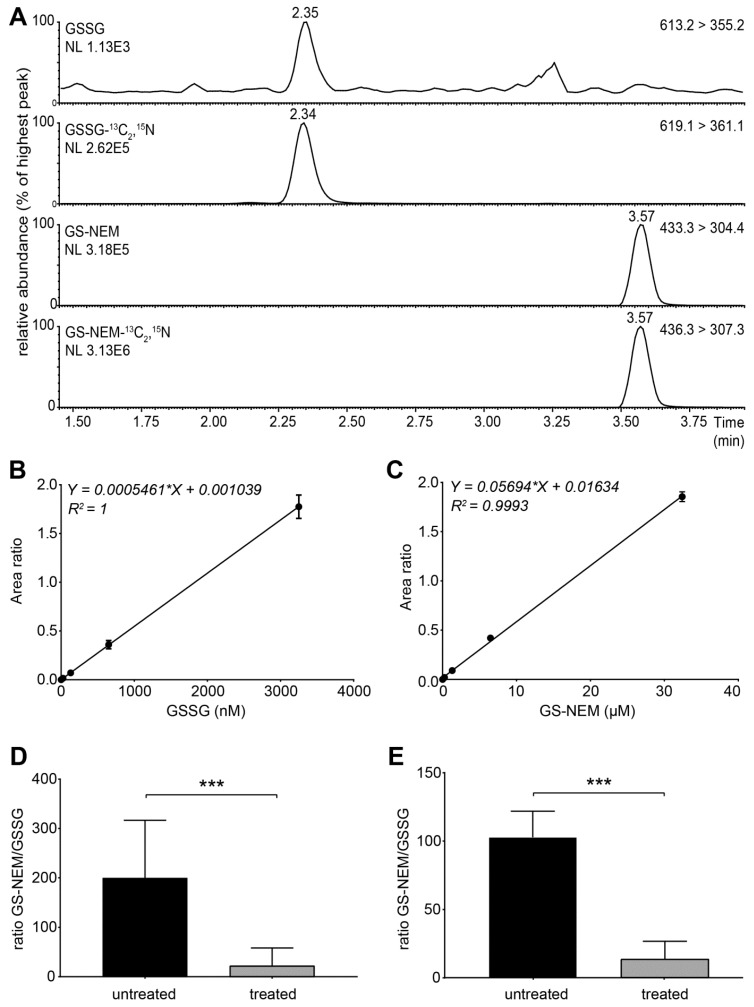
Method validation. (**A**) Representative extracted ion chromatograms of glutathione in an oxidised form (GSSG), GS-NEM and their isotopically labelled internal standards from control cells prepared using the presented sample preparation and UPLC-MS/MS method are shown. Mass transitions and the signal intensity (NL) are indicated for each analyte. (**B**,**C**) Calibration curves were generated using serially diluted amounts of unlabelled analyte for (**C**) GSSG (0–3.25 µM), and (**D**) GSH-NEM (0–32.41 µM), and a constant amount of corresponding isotopically labelled internal standard. Each data point represents the mean area ratio ± standard deviation duplicated measurements. Invisible error bars fall within the symbol size. Linearity was determined by linear regression, and the coefficient of determination (R2) was used as a goodness of fit. (**D**,**E**) GS-NEM/GSSG ratio in cells incubated with (**D**) 25 µM menadione for 1 h or (**E**) 12.5 µM BSO for 24 h, or vehicle. Data present the GSH/GSSG ratio. Additional plots of analytes are presented in Figure S1. Data are shown as bar plots with standard error. Mann–Whitney U test was performed to determine significant differences between the groups (*** *p*-value < 0.001).

1. Seed cells in 6-well plates the day prior to the experiment.
  Important: seed the cells in order to reach > 50% confluency on the day of the experiment.
2. **OPTIONAL STEP** On the next day, remove the culture medium of the cultured cells and add medium containing the chemicals of interest to the cells. For our experiments (see Figure 2 for details), we prepared stock solutions of BSO in Milli-Q water, stock solutions of menadione in ethanol, and stock solutions of me-C26:0 in DMSO. The methyl ester of C26:0 (me-C26:0) was prepared as described previously [7]. Incubate the cells for the desired time.
  Important: the maximum volume of added solvent may not exceed 1% *v/v* of the cell culture medium. As control, incubate cells with vehicle only, i.e., using an equal amount of solvent as was used for the incubations with the chemicals of interest.
3. Take the cultured cells from the incubator and place the 6-well plate on ice.
4. Remove the cell culture medium quickly and thoroughly, e.g., by suction.
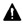
**CRITICAL STEP** Add ice-cold precipitation solution and internal standard immediately to avoid auto-oxidation. Carefully shake the plate to make sure the entire surface of the well has been covered.
6. Harvest the cells by scraping using a cell scraper.
7. Collect both cells and supernatant in a 2.0 mL microcentrifuge tube.
8. Centrifuge the samples at 5000× *g* for 10 min at 4 °C.
9. Transfer the supernatant to a new microcentrifuge tube.
10. Evaporate the supernatant under a nitrogen stream at 45 °C (duration ca. 00:30 hours).
11. Re-suspend the pellet in 100 µL of mobile phase A.
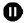
**PAUSE STEP** The sample can be stored at −20 °C until analysis.
13. Dry the cell pellet at room temperature.
14. Re-suspend the cell pellet in 200 µL NaOH in Milli-Q water (0.2 M concentration).
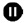
**PAUSE STEP** The sample can be stored at −20 °C until analysis.
16. Determine the protein concentration of the cell pellet, e.g., using the bicinchoninic acid assay [8].

### 3.3. Incubation and Preparation of S. cerevisae. Time for Completion: 02:00 Hours

1. On the day prior to the experiment, shift the yeast cells from minimal ethanol growth medium (see Section 2.3 for details) to oleate/ethanol growth medium, consisting of 25 mM potassium phosphate (pH = 6.0), 3 g/L yeast extract, 5 g/L Peptone, 3.2 g/L oleate solution (12 g oleate + 20 mL tween-80), and 20 g/L ethanol.
  Important: allow the cells to grow in minimal medium for at least 24 h before switching to oleate/ethanol medium. Incubate for 17 h at 28 °C.
2. Determine OD at 600 nm.
3. Take 1 mL of yeast culture with OD = 1.0 and add 300 µL of wash solution (200 mM NEM).
4. Mix briefly by vortexing or shaking.
5. Centrifuge at 7000 rpm for 1 min at 4 °C.
6. Remove the supernatant.
7. Add 1 mL of wash solution (50 mM NEM) to the cell pellet.
8. Vortex for 10 s.
9. Centrifuge at 7000 rpm for 1 min at 4 °C.
10. Repeat step 7–10 for a second wash.
11. Remove the supernatant.
12. Add ice-cold precipitation solution and internal standard.
13. Add glass beads.
14. Vortex for 5 min at 4 °C.
15. Centrifuge at 7000 rpm for 1 min at 4 °C.
16. Transfer the supernatant to a new microcentrifuge tube.
17. Evaporate the supernatant under a nitrogen stream at 45 °C (duration ca. 00:30 hours).
18. Re-suspend the pellet in 100 µL of mobile phase A.
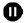
**PAUSE STEP** The sample can be stored at −20 °C until analysis.

### 3.4. UPLC-MS/MS Measurement. Time for Completion: 01:00 Hours

1. Use 10 µL of sample to inject onto a UPLC HSS T3 1.8 µm column (2.1 × 100 mm) using a UPLC system.
2. Set the column temperature to 50 °C and the flow rate to 400 µL/min.
3. Use the following chromatographic conditions: 99.9% A to 2% B/98% A in 2 min., 2–3.5 min. to 30% B/70% A, to 95% B/5% A in 0.1 min., 3.51–4.5 min. isocratic 95% B/5% A, to 99.9% A in 0.1 min. The equilibration time with solvent A is 1.5 min.
4. Use the triple-quadrupole mass spectrometer in positive electrospray ionization (ESI) mode.
5. Use nitrogen as nebulizing gas and argon as collision gas at a pressure of 2.5 × 10^3^ mbar.
6. Set the capillary voltage to 3.5 kV and the source temperature to 120 °C. Set the desolvation gas flow to 900 L/h and the desolvation temperature to 350 °C.
7. Set the instrument to multiple reaction monitoring (MRM) in the positive ionization mode to detect GSH-NEM*, GSSG and the labelled internal standards. Use the following transitions for detection: *m*/*z* 311 > 165 (GSH-^13^C_2_,^15^N), *m/z* 308 >162 (GSH), *m/z* 433 > 304 (GS-NEM), *m/z* 436 > 307 (GS-NEM-^13^C_2_,^15^N), *m/z* 613 > 355 (GSSG)**, and *m/z* 619 > 361 (GSSG-^13^C_4_,^15^N_2_) (Figure S2). *NEM reacts with free sulfhydryl groups and thus forms a derivative with GSH molecules (GS-NEM). Accordingly, we detected the ^13^C, ^15^N-labelled internal standard for GSH as GS-NEM (Figure 2A). **For GSSG, we detected both singly and doubly charged molecular ions. The concentrations of GSSG calculated based on both ions were similar, but the formation of doubly charged GSSG ions was less reproducible in several experiments (data not shown). Therefore, we used the singly charged ion as precursor for quantification of GSSG (Figure 2A).

## 4. Results

### 4.1. Assay Validation and Data Analysis

The use of stable isotope-labelled internal standards and hyphenated UPLC-MS/MS in combination with multi reaction monitoring (MRM) allowed for highly selective measurement of each analyte (Figure 2A). We integrated peaks using the Masslynx V4.1 software. We generated calibration curves using serially diluted amounts of unlabelled analytes for GSH-NEM and GSSG and a constant amount of the corresponding isotope-labelled internal standards (Figure 2B,C). To determine the concentration of GSH and GSSG in the sample, we divided the peak area of the compound peak by the peak area of the corresponding internal standard, i.e., GSSG (613.2 > 355.2)/GSSG-^13^C_2_,^15^N (619.1 > 361.1) and GS-NEM (433.3 > 304.4)/GS-NEM-^13^C_2_,^15^N (436.3 > 307.3), respectively. Subsequently, we divided the obtained value by the slope of the corresponding calibration curve. Finally, we divided the obtained concentration for GSH and GSSG, respectively, in the sample by the protein concentration of the cell pellet (in mg/mL) to adjust for differences in fibroblast cell confluency per culture well.

For the determination of linearity, we performed a serial dilution of resuspended fibroblast pellets, using Milli-Q as diluent. The limit of quantification (LOQ) was determined as signal-to-noise (S/N) ratios > 10, the limit of detection (LOD) was determined as an S/N ratio > 3. The limit of quantification (LOQ; signal to noise (S/N) ratio >10) was 0.33 nmol for GSSG, and 1.50 nmol for GS-NEM. The limit of detection (LOD; S/N ratio >3) was 0.10 nmol for GSSG, and 0.45 nmol for GS-NEM, and the S/N ratios for the respective peaks was greater than 10.

### 4.2. Applying the Method to Cells Exposed to Exogenous and Endogenous Stress Inducers

We investigated the effect of oxidative stress inducers on the cellular gluthatione-redox state. For this, we used primary control fibroblasts and cells with different metabolic defects. First, we used two chemical compounds, menadione and buthionine sulfoximine (BSO), to study the glutathione levels in control fibroblasts with and without an oxidative challenge. Menadione increases ROS production by redox cycling, and high concentrations of menadione have been reported to induce apoptosis [9]. BSO depletes the glutathione pool by inhibition of γ-glutamylcysteine synthetase, the first enzyme in glutathione synthesis [3]. Incubation of cells with low levels of menadione resulted in a significant decrease of the GSH/GSSG ratio, and the GSSG levels were significantly increased in cells incubated with 25 µM of menadione for 1 h (Figure 2D; Figure S1A–D). Similarly, incubation with 12.5 µM BSO for 24 h resulted in depleted levels of GSH and GSSG (Figure S1E–H) and the GSH/GSSG ratio was significantly decreased (Figure 2E).

**Figure 3:**
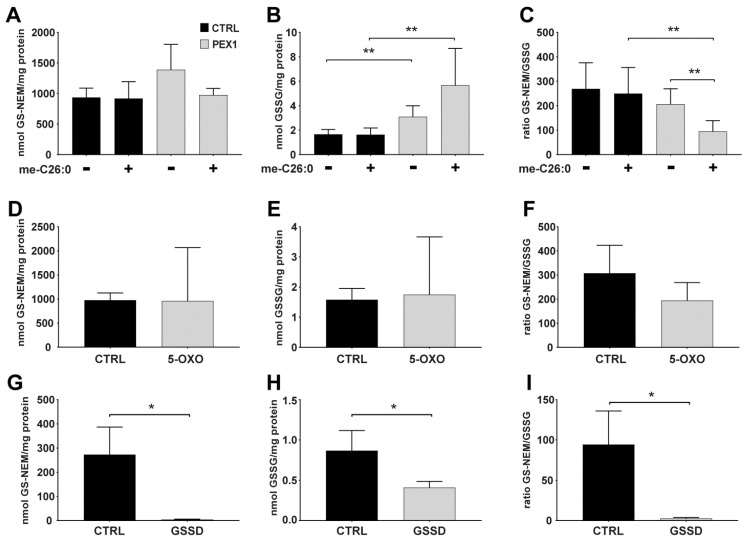
Glutathione levels in PEX1-deficient fibroblasts and cells with defects in glutathione metabolism. (**A**–**C**) Control and Pex1-deficient fibroblasts were incubated with methylated cerotic acid (me-C26:0) for 96 h, or with DMSO as vehicle. (**D**–**I**) Glutathione levels of (**D**–**F**) 5-oxoprolinase-deficient fibroblasts (5-OXO), and (**G**–**I**) glutathione synthetase-deficient fibroblasts (GSSD). (**A**,**D**,**G**) GS-NEM, (**B**,**E**,**H**) GSSG, and (**C**,**F**,**I**) ratio GS-NEM/GSSG. Data are shown as bar plots with standard error. Mann–Whitney U test was performed to determine significant differences between the groups (* *p*-value < 0.05; ** *p*-value < 0.01).

Next, we investigated the effect of accumulating very long-chain fatty acids in metabolically disturbed cells. To this end, we used primary fibroblasts with a mutation in the *PEX1* gene. In these cells, peroxisome biogenesis is disturbed, and cells accumulate very long-chain fatty acids such as cerotic acid (C26:0), because of a defective peroxisomal fatty acid ß-oxidation. When we incubated PEX1-deficient fibroblasts with 40 µM of me-C26:0 for 96h, we detected normal concentrations of GSH, while GSSG concentrations were significantly increased when compared to untreated PEX1-deficient fibroblasts (Figure 3A,B). When compared to control cells incubated with cerotic acid, the GSSG concentrations were also increased in PEX1-deficient fibroblasts. Consequently, the GSH/GSSG ratio was decreased in PEX1-deficient cells incubated with cerotic acid when compared to untreated PEX1-deficient cells, as well as when compared to treated control cells (Figure 3C). Treatment with cerotic acid had no influence on the GSH/GSSG ratio in control cells (Figure 3A–C).

### 4.3. Glutathione Levels in Cultured Cells with Different Genetic Defects in Glutathione Metabolism

We next investigated glutathione levels in primary fibroblasts with a deficiency of 5-oxoprolinase and glutathione synthetase, respectively. These enzymes are involved in the γ-glutamyl cycle, and a deficiency of either enzyme causes pyroglutamic aciduria (5-oxoprolinuria) in patients [10]. We determined GSH and GSSG concentrations in fibroblasts deficient of these enzymes and compared them to control cells. In 5-oxoprolinase-deficient fibroblasts, concentrations of both GSH and GSSG were normal, and the GSH/GSSG ratio was not changed when compared to control cells (Figure 3D–F). In glutathione synthetase-deficient cells, however, GSH levels were depleted, and the concentrations of GSSG were significantly decreased when compared to control fibroblasts. (Figure 3G,H). Accordingly, the GSH/GSSG ratio was substantially reduced in these cells (Figure 3I).

### 4.4. Glutathione Levels in the Yeast S. cerevisiae

To determine the applicability of our method to other cell types, we also measured glutathione levels in the yeast *S. cerevisiae*. To this end, we modified the sample preparation protocol by already including NEM in the wash buffer when harvesting the yeast cells in order to immediately block thiol groups. As a proof-of-principle, we determined the levels of GSH and GSSG in wild-type yeast and in a mutant strain lacking the *glr1* gene, which encodes glutathione reductase. This enzyme is crucial for the reduction of GSSG to GSH, and yeast mutants lacking *glr1* (glrΔ) have been described to have increased levels of GSSG [11]. Using our assay, concentrations of GSSG, but also GSH were significantly increased in the mutant strain (Appendix A). The GSH/GSSG ratio was significantly decreased in the mutant strain when compared to the wild-type strain (Appendix A).

## 5. Reagents Setup

We prepared labelled oxidised glutathione-(glycine-^13^C_2_,^15^N) by oxidation using potassium iodide and hydrogen peroxide [12]. In brief, labelled reduced glutathione-(glycine-^13^C_2_,^15^N) was dissolved in Milli-Q water (stock concentration: 6.5 mM), and incubated with a solution of potassium iodide in Milli-Q water (final concentration: 0.67 mM) and hydrogen peroxide (30%) for 120 min at 50 °C, followed by incubation for 5 min at 65 °C to inactive hydrogen peroxide activity. We prepared stock solutions of labelled standards in Milli-Q water, and stored samples at −20 °C.

## Figures and Tables

**Figure A1:**
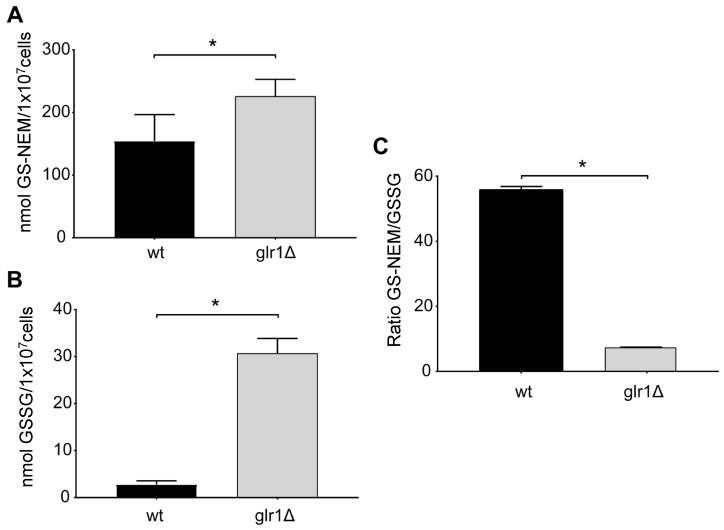
Glutathione levels in the yeast *S. cerevisiae*. Glutathione levels of WT and glr1 deletion strains are shown as bar plots with standard error. (**A**) GS-NEM, (**B**) GSSG, and (**C**) ratio GS-NEM/GSSG. Mann–Whitney U test was performed to determine significant differences between the groups (* *p*-value < 0.05).

## Supplementary Materials

The following are available online at https://www.mdpi.com/2218-1989/9/3/45/s1: Figure S1: Effect of menadione and BSO on GS-NEM and GSSG levels in fibroblasts; Figure S2: Fragmentation pattern of GS-NEM and GSSG.

## Appendix B

Comparison of results with those obtained by other LC-MS-based protocols. We compared the levels of GSH and GSSG presented in this study to concentrations of GSH and GSSG determined by HPLC methods in cultured cells and physiological fluids reported in the literature. In a study using NEM and trichloroacetic acid for sample preparation of various human and bovine cell lines, the reported concentrations of both GSH and GSSG were approximately one order of magnitude lower than we measured with our assay [4]. The GSH/GSSG ratio, however, was comparable to the levels reported in our study [4]. In two other studies using NEM and sulfosalicylic acid for the preparation of whole blood samples from humans, and from a mouse model with chronic oxidative stress, glutathione levels and the GSH/GSSG ratio were similar in blood samples when compared to cultured cells measured with the method presented in the present article [13,14]. In a study using NEM and acetonitrile for the preparation of blood and saliva samples, the levels of GSH and GSSG in red blood cells exceeded the GSH concentrations in cultured fibroblasts we measured [5]. In saliva, the GSH concentrations were similar when compared to cultured fibroblasts, whereas the concentrations of GSSG were much higher [5]. Since Fahrenholz et al. [5] used a similar approach for sample preparation as presented in our paper, the differences in GSH and GSSG concentrations are likely due to the different concentrations present in the red blood cells and saliva when compared to cultured fibroblasts. It has been reported that tissues and cells have lower glutathione concentrations when compared to physiological fluids [3].
