# Supplementary material for: An UPLC-MS/MS Assay to Measure Glutathione as Marker for Oxidative Stress in Cultured Cells
Source: Metabolites. 2019 Mar 5;9(3):45. doi: 10.3390/metabo9030045 (PMC6468779; doi:10.3390/metabo9030045)

Figure S1: Effect of menadione and BSO on GS-NEM and GSSG levels in fibroblasts. Control fibroblasts were incubated with A, B) different concentrations of menadione for 1 h, C, D) 25 µM of menadione for 1h, E, F) different concentrations of BSO for 24 h, or G,H) 12.5 µM of BSO for 24h. A, C, E, G) GS-NEM, and B, D, F, H) GSSG levels. Data in A, B, E, and F are shown as line chart, and each data point is indicated by a dot. Data in C, D, G, and H are shown as bar plots with standard error. Mann–Whitney U test was performed to determine significant differences between the groups (** p-value < 0.001; *** p-value < 0.001).

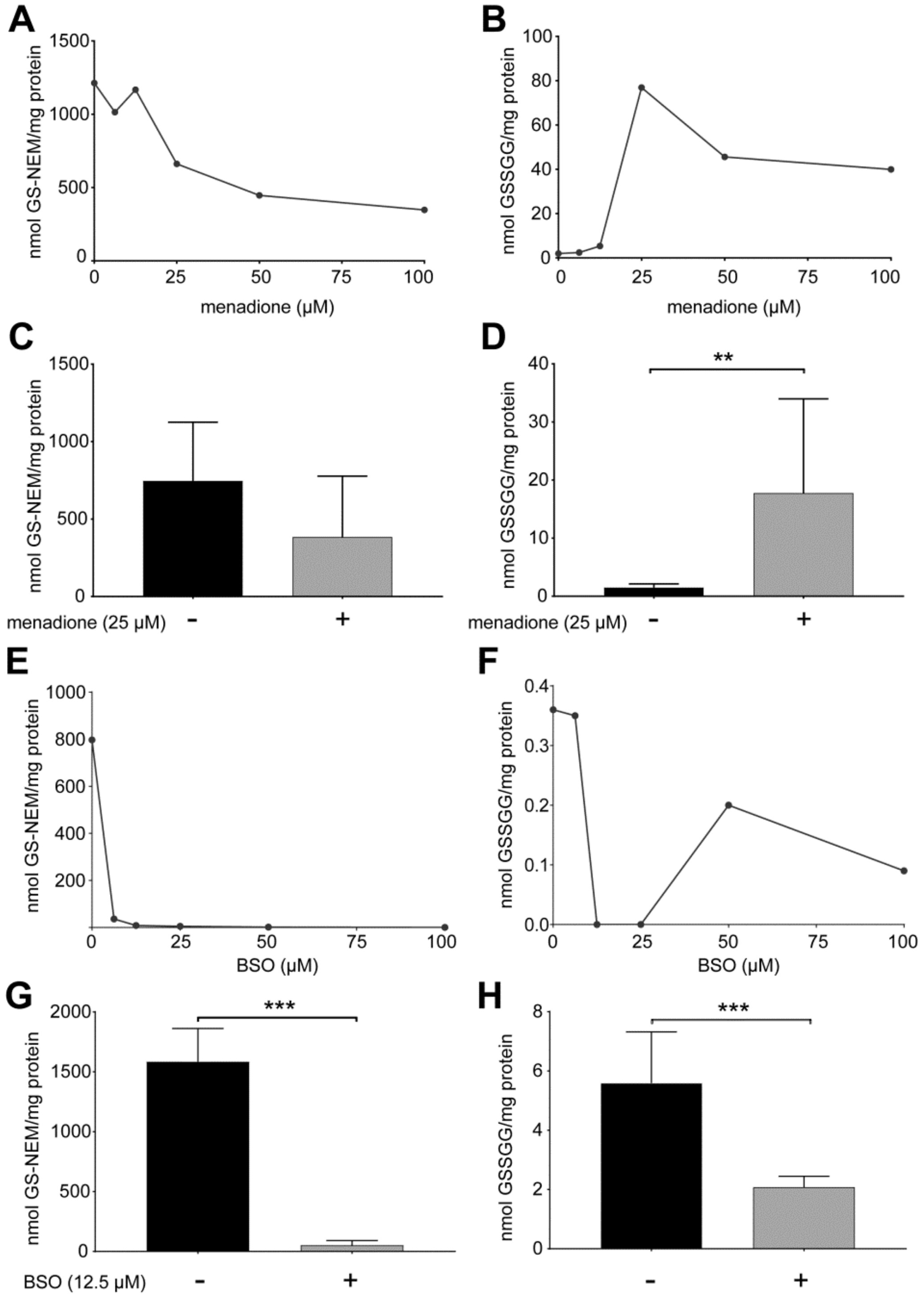

Figure S2: Fragmentation pattern of GS-NEM and GSSG.

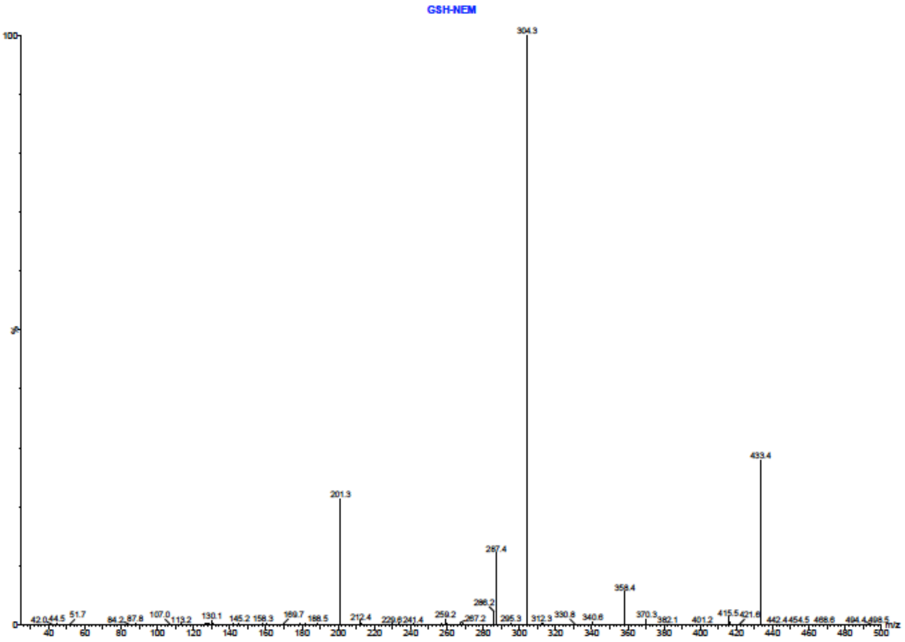

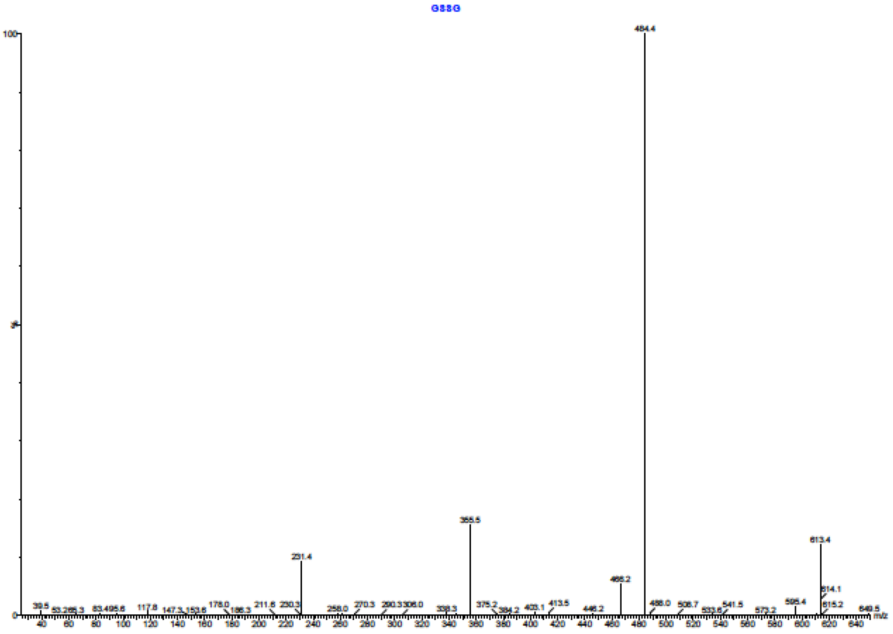

Supplement: Supplementary file 1 [file metabolites-09-00045-s001.pdf]
